# Multimodal Contrast Agents for Optoacoustic Brain Imaging in Small Animals

**DOI:** 10.3389/fbioe.2021.746815

**Published:** 2021-09-28

**Authors:** Xue-feng Shi, Bin Ji, Yanyan Kong, Yihui Guan, Ruiqing Ni

**Affiliations:** ^1^ Department of Respiratory Medicine, Qinghai Provincial People’s Hospital, Xining, China; ^2^ Department of Radiopharmacy and Molecular Imaging, School of Pharmacy, Fudan University, Shanghai, China; ^3^ PET Center, Huashan Hospital, Fudan University, Shanghai, China; ^4^ Institute for Regenerative Medicine, University of Zurich, Zurich, Switzerland; ^5^ Institute for Biomedical Engineering, University of Zurich and ETH Zurich, Zurich, Switzerland

**Keywords:** optoacoustic (photoacoustic) imaging, animal model, brain imaging, multimodal imaging, contrast agent, fluorescence imaging, nanoparticle, magnetic resonance imaging

## Abstract

Optoacoustic (photoacoustic) imaging has demonstrated versatile applications in biomedical research, visualizing the disease pathophysiology and monitoring the treatment effect in an animal model, as well as toward applications in the clinical setting. Given the complex disease mechanism, multimodal imaging provides important etiological insights with different molecular, structural, and functional readouts *in vivo*. Various multimodal optoacoustic molecular imaging approaches have been applied in preclinical brain imaging studies, including optoacoustic/fluorescence imaging, optoacoustic imaging/magnetic resonance imaging (MRI), optoacoustic imaging/MRI/Raman, optoacoustic imaging/positron emission tomography, and optoacoustic/computed tomography. There is a rapid development in molecular imaging contrast agents employing a multimodal imaging strategy for pathological targets involved in brain diseases. Many chemical dyes for optoacoustic imaging have fluorescence properties and have been applied in hybrid optoacoustic/fluorescence imaging. Nanoparticles are widely used as hybrid contrast agents for their capability to incorporate different imaging components, tunable spectrum, and photostability. In this review, we summarize contrast agents including chemical dyes and nanoparticles applied in multimodal optoacoustic brain imaging integrated with other modalities in small animals, and provide outlook for further research.

## Introduction

### Optoacoustic Imaging

The multimodal imaging strategy across different scales has gained huge interest in recent years. The advances in neuroimaging such as positron emission tomography (PET) and magnetic resonance imaging (MRI) have provided valuable tools for understanding brain function, for early and differential diagnosis of brain disorders and for monitoring treatment effect (Fox and Raichle, 2007; Langen et al., 2017; Hansson, 2021; Kreisl et al., 2021). Optoacoustic (photoacoustic; OA) imaging is an emerging imaging tool and has demonstrated versatile applications in biomedical research (Deán-Ben et al., 2016; Knieling et al., 2017; Masthoff et al., 2018; Neuschmelting et al., 2018; Gottschalk et al., 2019; Qian et al., 2019; Karlas et al., 2021; Na et al., 2021; Razansky et al., 2021). OA imaging utilizes absorption of light as a source of contrast, while the emitted ultrasound (US) is used for image formation (Razansky et al., 2009; Wang and Yao, 2016). As the spatial resolution of OA imaging is not changed by photon scattering, it thus exhibits a unique combination of high sensitivity and high spatial resolution. The detection depth of OA ranges from millimeters to centimeters, which associates with spatial resolution (from <1 μm in OA microscopy to 100 μm in OA tomography) (Li et al., 2017; Zhang et al., 2019a; Li et al., 2020). Recent OA tomography has allowed imaging the whole mouse brain with <100 µm spatial resolution *in vivo* (Deán-Ben et al., 2016; Vaas et al., 2017; Gottschalk et al., 2019; Ni et al., 2020a; Ni et al., 2021) which is around 10 times higher than the resolution achievable by using commercial small-animal microPET scanners (Lancelot and Zimmer, 2010).

### Multimodal Optoacoustic Brain Imaging

Assessing the brain function under whole brain complex network dynamics is the key for understanding physiology of the brain and deciphering brain disorders. While PET provides excellent accuracy in quantification, the limited spatial resolution (approximately 1 mm) relative to the small mouse brain and the signal spillover hinders accurate mapping of the target (Lancelot and Zimmer, 2010). Optical two-photon microscopy offers excellent spatial resolution, however limited by depth penetration and small (sub-mm) field-of-view (Ntziachristos, 2010). Macroscopic imaging with MRI provides high resolution however has the limitations in sensitivity and temporal resolution (Razansky et al., 2021). The use of hybrid contrast agents in multimodal imaging has enabled to detect the targets with different sensitivity and provide comprehensive molecular information, as well as better soft tissue contrast, facilitating accurate quantification (e.g., combined with MRI and computed tomography (CT) (Ren et al., 2019). Various multimodal OA molecular imaging techniques have been applied in preclinical brain imaging studies, including OA/fluorescence imaging, OA/MRI, OA/US, OA/MRI/Raman, OA/PET, OA/single-photon emission computerized tomography (SPECT), and OA/CT. (Kircher et al., 2012; Fan et al., 2014; Zhu et al., 2015; Qiao et al., 2018) (Table 1).

**TABLE 1:** Summary of hybrid contrast agents for multimodal optoacoustic brain imaging.

Modality	Contrast agent	Abs	Target
OA/FL	AOI987 Ni et al. (2020a)	650	Amyloid-β, AD
	CRANAD-2 Ni et al. (2021)	640	
	Congo red Zhou et al. (2021)	500	
	PBB5 Vagenknecht et al. (2021)	635	Tau, AD, FTD
	CDnir7 Park et al. (2019)	806	Microglia, macrophage, AD
	IRDye 800CW–conjugated CAIX-800 Huang et al. (2020)	800	Hypoxia, nasopharyngeal tumor
	MMPsense Ni et al. (2018a)	680	MMP, stroke
	Cu_2_-xSe NPs Zhang et al. (2019b)	NIR II	ROS, glioblastoma
	Conjugated polymer NP Guo et al. (2017)	NIR II	Glioblastoma
	Semiconducting polymer NP Yang et al. (2019)	NIR II	
	Cu_2_-xSe NPs, DOX‐HCu Wu M. et al., (2018)	808	
	Single-layer MoS2 Nanosheets Chen et al. (2016)	675	
	P1RGD NP Guo et al. (2018)	NIR II	
	PBT NP Guo et al. (2019a)	NIR II	
	PTD NP Guo et al. (2019b)	NIR II	
	IRDye800-H-ferritin NP Jia et al. (2020)	800	
	Aggregation-induced emission dots Sheng et al. (2018)	NIR II	
	CR780RGD-NPs Liu et al. (2021b)	780	
	ICG/AuNR@BCNP Yang et al. (2020)	808	
	ICG-holo-transferrin NP Zhu et al. (2017)	780	
	SPN-OT, SPN-PT, and SPN-DT Jiang et al. (2019)	NIR II	Metabolizable
	QC-1/BSA/BODIPY Cardinell et al. (2021)	750	Lymphatic drainage
	Prussian blue particle–labeled MSC Li et al. (2018a)	701	Brain injury
	Polymer-blend dot-chlorotoxin Wu et al. (2011)	488	Medulloblastoma
OA/SPECT	CPMSN@[^125^I]SD Yao et al. (2020)	680	MSC, stroke
OA/SPECT/FL	[^131^I]A1094@RGD-HBc Liu et al. (2019a)	NIR II	Glioblastoma
	[^99^mTc]UCS Zhang et al. (2018a)	633	Blood–brain barrier
OA/PET/FL	[^18^F]CDA-3 Liu et al. (2017)	798	Amyloid-β, AD
OA/PET	[^64^Cu]RGD-Au-tripod Cheng et al. (2014)	710	Glioblastoma
OA/PET/MRTI	[^64^Cu]c(KRGDf)-PEG-HAuNS Lu et al. (2011)	800	
OA/PET/MRI/FL	IRDye78-α-LA-DFO-[^89^Zr] Yang et al. (2021)	770	
OA/MRI	Prussian blue–poly(l-lysine) NP Kim et al. (2017)	715	MSC
	Prussian blue nanocubes (PBNCs) Kubelick and Emelianov, (2020)	734	MSC, spinal cord
	Magneto-plasmonic MNP@Au nanostars Tomitaka et al. (2020)	710	Drug delivery
	gM-Luc-GRMNBs Chen et al. (2015)	810	MSC, stroke
	SPIO@Au-labeled MSC Qiao et al. (2018)	810	MSC
	Gd-PEG-polypyrrole NPs Liang et al. (2015)	808	Glioblastoma
	cRGD-CM-CPIO Duan et al. (2020a)	730	
	HALF-cRGD Duan et al. (2020b)	685	
	Mn^2+^-doped Prussian blue Zhu et al. (2015)	808	
OA/CT/MRI	Core-shell Au nanorod@metal-organic NP Shang et al. (2017)	720	
OA/SWIR/CT/UCL	NaErF_4_:Tm@NaYF_4_:Yb@NaLuF_4_:Nd,Yb-ZnPc Lv et al. (2019)	808	
OA/US	PDI NP Fan et al. (2015)	700	
OA/Raman	SERRS-MSOT-nanostar Neuschmelting et al. (2018)	770	
OA/MRI/Raman	Maleimide-DOTA-Gd @Au-silica–based SERS Kircher et al. (2012)	540	

Abs, absorbance; AD, Alzheimer’s disease; Au, gold; CT, computed tomography; FL, fluorescence imaging; FTD, frontotemporal dementia; Gd, gadolinium; MRI, magnetic resonance imaging; MRTI, magnetic resonance thermal imaging; MSC, mesenchymal stem cells; MMP, matrix metalloproteinases; NP, nanoparticle; OA, optoacoustic imaging; PDI, perylene-diimide; PEG, polyethylene glycol; PET, positron emission tomography; ROS, reactive oxygen species; SPECT, single-photon emission computed tomography; SPIO, superparamagnetic iron oxide; SWIR, short-wavelength infrared; US, ultrasound imaging; UCL, upconversion luminescence;

## Hybrid Contrast Agents for Multimodal OA Brain Imaging

The contrast of OA imaging comes from endogenous tissue contrasts or chromophores (e.g., oxyhemoglobin (HbO)/deoxyhemoglobin (Hb), melanin, and lipids), as well as from the administrated spectrally distinctive exogenous contrast agents (Weber et al., 2016). The majority of preclinical OA molecular imaging in the brain has been focused on detecting the pathological changes in a glioblastoma model, and applications have also emerged in animal models of stroke, epilepsy, Alzheimer’s disease (AD), and neuroinflammation (Ni et al., 2017; Xi et al., 2017; Ni et al., 2018a; Ni et al., 2018b; Ishikawa et al., 2018; Ni et al., 2020a; Kasten et al., 2020; Razansky et al., 2021). Different types of exogenous contrast agents have been developed, including synthetic (chemical dyes or nanoparticles (NPs)), semi-genetic, and genetic contrast agents (e.g., genetically encoded calcium indicators and reversibly switchable OA proteins (Roberts et al., 2018; Qian et al., 2019; Mishra et al., 2020; Farhadi et al., 2021; Qu et al., 2021; Shemetov et al., 2021)). The criteria for contrast agent applied in OA brain imaging include a suitable absorbance spectrum (>600 nm wavelength) to allow unmixing with endogenous signals (e.g., Hb/HbO and melanin) and sufficient brain penetration depth, high affinity and specific binding to the target, sufficient blood–brain barrier entrance, photostability, solubility, low toxicity, high thermodynamics for MRI probes, and optimal pharmacokinetics (Weber et al., 2016). Chemical dyes are mainly used for OA/fluorescence imaging and have the advantage of low toxicity, sufficient blood–brain barrier entrance due to the small molecular weight, fast metabolism, and clearance; however, they have limited adjustment potential. The NPs utilized for OA imaging are mainly carbon-based NPs, for example, single-walled carbon nanotubes; metal-based NPs, for example, gold NPs; bismuth-based NPs; polymer-encapsulated organic NPs; semiconducting polymer NPs (SPNs); conjugated polymer; and novel DNA-based nanocarriers. (Pu et al., 2014; Li and Chen, 2015; Weber et al., 2016; Yang et al., 2018; Yu et al., 2019; Zhan et al., 2019; Xu et al., 2020; Cheng et al., 2021; Fan et al., 2021; Joseph et al., 2021; Qi et al., 2021a; Tuo et al., 2021; Wang et al., 2021a; Wang et al., 2021b; Zhen et al., 2021). NPs have the advantage of versatile multimodal imaging capacity, a favorable signal/noise ratio, high photothermal conversion, deep penetration depth with near-infrared (NIR) II probes, and diverse structure and types (activable, turnable, and metabolizable). However, the stability, biodegradability, biocompatibility, clearance toxicity, nanostructural control, and blood–brain barrier entrance of NPs require careful designing (Liu et al., 2021a).

### OA/Fluorescence Imaging

#### Chemical Dyes

Many chemical dyes for OA imaging have fluorescence properties and have been widely used in hybrid OA/fluorescence imaging (Li et al., 2021a); the OA/fluorescence dye ideally has a distinct absorption peak and a relatively low quantum yield to allow OA detection, for example, IRDye 800CW (Attia et al., 2016) and naphthalocyanine (Bézière and Ntziachristos, 2015), indocyanine green (ICG) (Mokrousov et al., 2021), and Prussian blue. Administration of ICG visualizes blood vessels and enables OA/fluorescence imaging of cerebral perfusion in glioblastoma mouse models (Burton et al., 2013). Neuroinflammation and glial activation–related molecular changes are implicated in many brain disorders, such as stroke, multiple sclerosis, and AD (Leng and Edison, 2021; McAlpine et al., 2021). The change in the levels of endogenous oxygen saturation (calculated based on hemoglobin readouts) has been used as an indicator for neuroinflammation in rats with stereotaxic injection of lipopolysaccharides (LPS) (Guevara et al., 2013). Targeted probes for molecular changes including matrix metalloproteinases and nitric oxide production have been employed for visualizing neuroinflammation in animal models (McQuade et al., 2010; Qi et al., 2021b). Upregulated levels of matrix metalloproteinases (MMPs) were detected using an MMPsense probe (e.g., 680 nm) with OA/fluorescence imaging approaches in the cerebral ischemic lesion region of a mouse model at 48 h after transient middle cerebral artery occlusion (Ni et al., 2018a). In addition, recent OA/fluorescence imaging studies reported using NIR cyanine derivative CDnir7 to detect microglia and astroglia activation in the brain of triple transgenic AD mice (Park et al., 2019). CDnir7 has previously been utilized to detect macrophage uptake in the peripheral organs using fluorescence molecular tomography and OA tomography (Kang et al., 2014).

The cerebral accumulation and spreading of proteiopathies are central to neurodegenerative diseases including AD and Parkinson’s disease. Previous studies have utilized two-photon imaging and near-infrared imaging with probes BF-158, BODIPY derivative, HS-84, HS-169, methoxy-X04, and fluorescent-labeled antibodies (Krishnaswamy et al., 2014; Kuchibhotla et al., 2014; Verwilst et al., 2017; Wu Q. et al., 2018; Calvo-Rodriguez et al., 2019; Detrez et al., 2019; Voigt et al., 2019; Zhou et al., 2019; Fung et al., 2020; Ni et al., 2020b) for amyloid-β and tau detection at cellular resolution in animal models. Several studies have employed β-sheet binding OA/fluorescence hybrid dyes with an NIR range absorbance spectrum peak for *in vivo* imaging of the proteinopathy accumulation in the brain. OA tomography using oxazine derivative AOI987 has been shown to provide transcranial visualization of the bio-distribution of amyloid-β deposits in mouse models of AD amyloidosis (arcAβ and APP/PS1 model) (Ni et al., 2020a). A similar design using OA tomography with curcumin derivative CRANAD-2 in has been used in an arcAβ mouse model (Ni et al., 2021). OA microscopy with Congo red has been used for the detection of amyloid-β plaques and cerebral amyloid angiopathy in the APP/PS1 mouse model (Hu et al., 2009; Zhou et al., 2021). OA tomography with chemical dye PBB5 (PBB3 derivative) for detection of β-sheet–containing tau deposits in the P301L 4-repeat tau mouse model has been reported (Ono et al., 2017; Vagenknecht et al., 2021). It is foreseeable that OA tomography pipeline with the deep brain region detection capability will be applied together with OA/fluorescence β-sheet–binding dyes to image other proteiopathy disease models, such as Parkinson’s disease mouse model with α-synuclein accumulation and the amyotrophic lateral sclerosis animal model with TAR DNA-binding protein 43 deposits.

### Nanoparticles

NPs are widely used as hybrid contrast agents for their capability to incorporate different imaging components, tunable spectrum, and photostability. OA/fluorescence imaging has been reported for monitoring lymphatic drainage using QC-1/bovine serum albumin/BODIPY (Cardinell et al., 2021) and brain injury with Prussian blue particle–labeled mesenchymal stem cells (Li et al., 2018a). Many OA/fluorescence NPs are targeted toward integrin α(v)β(3) which is overexpressed in endothelial cells in the glioblastoma mouse model. Near-infrared (NIR) I range NPs in glioblastoma imaging include quantum dots (Zhao et al., 2020), gold NPs (Lu et al., 2011; Kircher et al., 2012; Lozano et al., 2012; Shang et al., 2017), copper/iron-based NPs (Wu M. et al., 2018; Zhou et al., 2018; Zhang et al., 2019b), carbon nanorods (Pramanik et al., 2009; Qian et al., 2018), MoS_2_ nanosheets (Chen et al., 2016; Guo et al., 2017), semiconducting polymeric NPs (Yang et al., 2019), nanodot–chlorotoxin conjugates (Wu et al., 2011), polymer-encapsulated organic NPs (Li and Liu, 2014), ICG-holo-transferrin NPs (Zhu et al., 2017), and liposomes (Miranda et al., 2019). The circulating dyes and NPs accumulate in brain tumors due to a disruption of the blood–brain barrier (Kircher et al., 2012; Burton et al., 2013; Neuschmelting et al., 2018) or enhanced permeability and retention effect (Li et al., 2018b). To enhance the brain uptake and OA signal, one strategy is to load the chemical dyes into NPs, for example, a recent study utilized CR780RGD-NPs, formed by conjugating the croconaine dye, NH_2_–polyethylene glycol (PEG) 2000-MAL, and the cancer-targeting c(RGDyC) peptide, to detect the tumor in the deep brain region in a glioblastoma mouse model (Liu et al., 2021b). Another strategy is to use activable hybrid OA/fluorescence probes for detection with higher specificity. The activable probes that have been reported mainly target at tumor-related hypoxia, glutathione, pH changes, and reactive oxygen species (Liu et al., 2021c). Hypoxia plays an important role in tumor metastasis and resistance to chemoradiotherapy and has been an important target for tumor imaging (Rankin and Giaccia, 2016). IRDye800-H-ferritin nanocarrier (IRDye800-HFn) (Jia et al., 2020) was applied in imaging hypoxia in glioma, and albumin-based gold (Au) NP, ICG/AuNR@BCNP, was used as theranostics for glioma- and hypoxia-alleviating treatment (Yang et al., 2020). In addition, an IRDye 800CW–conjugated probe CAIX-800 in imaging changes in carbonic anhydrase IX (CAIX) in nasopharyngeal carcinomas in a mouse model has been reported with excellent signal/noise ratios (Huang et al., 2020).

In the NIR I range, light scattering, hemoglobin absorbance, and skull attenuation interferes in the signal/noise ratio, unmixing, and penetration depth in the small animal brain (Wan et al., 2018; Liang et al., 2019). The skull attenuation positively associates with increasing age, which makes imaging in aged disease animal models difficult. Efforts are thus made to develop NIR II (>1,000 nm) hybrid OA/fluorescence probes (He et al., 2018; Huang and Pu, 2020; Dai et al., 2021). Several NIR II range NPs with excellent photothermal conversion efficiency have been applied for *in vivo* glioblastoma imaging in the mouse brain, for example, Cu_2_-xSe NPs for detecting reactive oxygen species (Zhang et al., 2019b), aggregation-induced emission dots A1094@RGD-HBc (Sheng et al., 2018), excitable semiconducting polymer NPs (Yang et al., 2019), and P1RGD NP conjugated polymers. (Guo et al., 2018). Jiang et al. (2019) reported metabolizable NIR II SPN for mouse brain imaging such as SPN-OT, SPN-PT, or SPN-DT of high photothermal conversion efficiencies and effective clearance with minimum toxicity.

### OA/Positron Emission Tomography; OA/Single-Photon Emission Computerized Tomography

**FIGURE 1:**
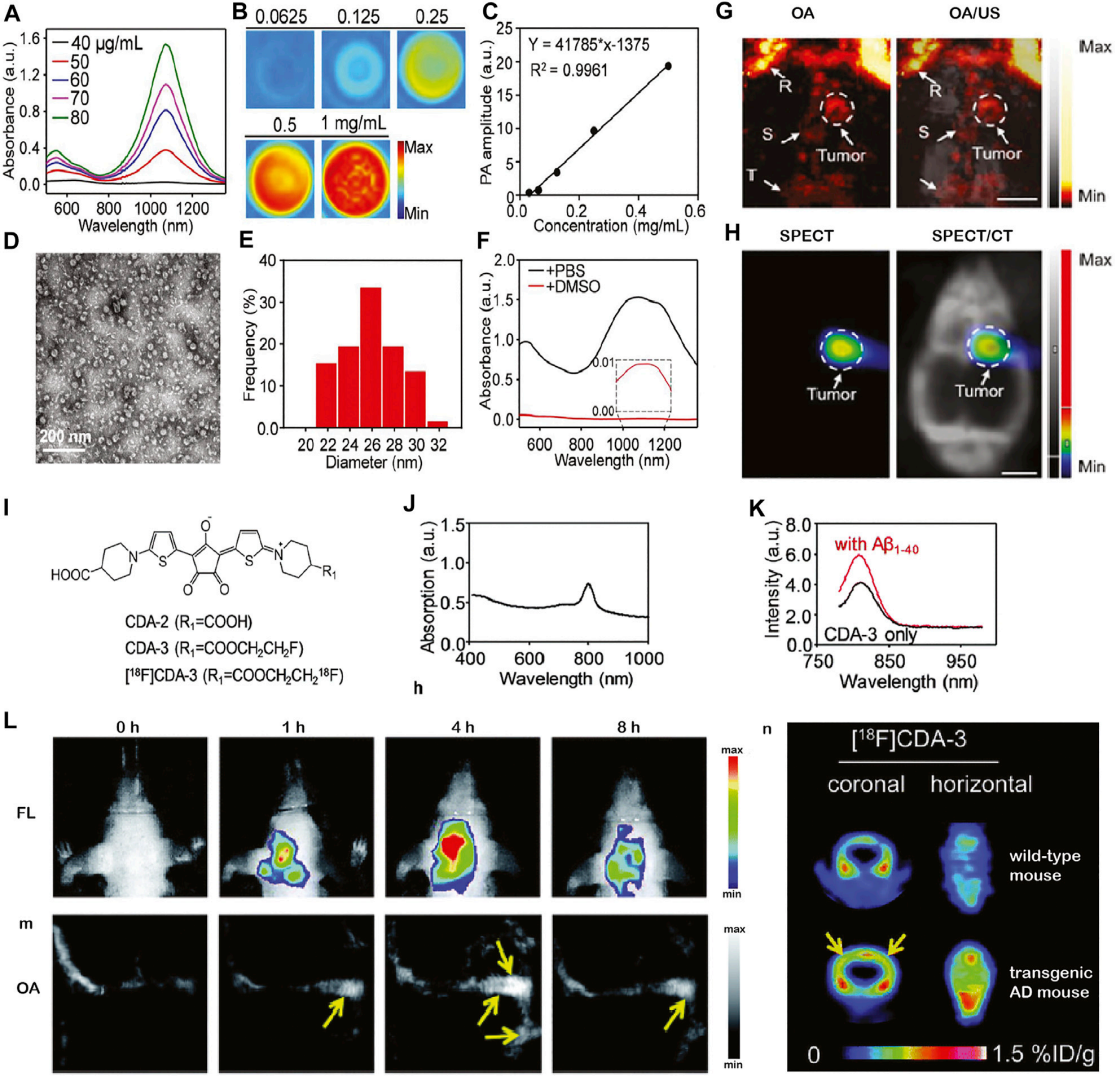
*In vivo* multimodal optoacoustic (OA) brain imaging in small disease animal models. **(A–H)** Multimodal optoacoustic (OA) imaging in the brain of U87MG tumor-bearing mice using [^131^I]A1094@RGD‐HBc. **(A–F)** Characterization of the A1094@RGD‐HBc; **(A)** absorption spectra of A1094; **(B)** OA images of A1094 at different concentrations at 950 nm in DMSO; and **(C)** relation between a OA signal and A1094 concentrations in DMSO. **(D)** Transmission electron microscope of A1094@RGD-HBc; **(E)** dynamic light scattering of A1094@RGD-HBc; **(F)** absorption spectra of A1094@RGD-HBc before/after 90% DMSO destroying the protein; **(G)** *In vivo* OA tomography/ultrasound images, and **(H)** microSPECT/CT images of the brain of U87MG tumor‐bearing mice at 2 h after injection of [^131^I]A1094@RGD‐HBc; Scale bar = 2 mm. R, rostral rhinal vein; S, sagittal sinus; T, transverse sinus; reproduced from ref. Liu et al. (2019a) with permission from the WILEY-VCH Verlag GmbH & Co. KGaA, Weinheim; **(I–N)** multimodal imaging of cerebral amyloid-β plaque in an Alzheimer’s disease mouse model. **(I)** Chemical structure of [^18^F]CDA-3; **(J)** UV-vis absorption curve of CDA-3; **(K)** fluorescence intensity of CDA-3 with/without Aβ1–40 aggregates; **(L–N)** *in vivo* OA tomography/positron emission tomography/near-infrared fluorescence imaging of brain amyloid-beta plaque detection in the Alzheimer amyloidosis mouse model. Reproduced from ref. Liu et al. (2017) with permission from the Royal Society of Chemistry.

Signal spillover is a known issue in small animal PET mainly due to the size of the small animal brain (Lancelot and Zimmer, 2010). The rational for integrating OA and PET/SPECT imaging is that OA imaging provides a higher resolution tomographic/or microscopic imaging, while PET/SPECT provides higher detection sensitivity. Several studies reported using single-walled carbon nanotubes at NIR II conjugated probes that target integrin for OA/PET or OA/SPECT tumor imaging in animal models, such as [^64^Cu]RGD-Au-tripod for OA/PET (Cheng et al., 2014) and [^131^I]A1094@RGD-HBc for OA/SPECT/CT in an U87MG tumor‐bearing glioblastoma mouse model (Liu et al., 2019a) (Figures 1A–H). The OA data correlated well with PET data as well as SPECT data in both studies. In addition to imaging brain tumor in animal models, OA/PET and OA/SPECT applications in AD and in the stroke mouse model as theragnostic agents have been reported (Liu et al., 2017; Yao et al., 2020). OA/PET/fluorescence triple-modality imaging of brain amyloid-β plaques has been demonstrated using functionalized croconium dye [^18^F]CDA-3 (Liu et al., 2017), showing cortical accumulation of amyloid-β deposits in mice with AD amyloidosis (Figures 1I–K). OA/SPECT imaging using CPMSN@[^125^I]SD, formed by cobalt protoporphyrin IX–loaded mesoporous silica NPs labeled with [^125^I]-conjugated/spermine-modified dextran polymer, was reported for tracking mesenchymal stem cells, exerting antioxidant effects, and improving the recovery in a mouse model of cerebral ischemia (Yao et al., 2020). Moreover, biodegradable ultrasmall Cu_2_–xSe NPs (diameter 3.0 nm) labeled with [^99^mTc] has been demonstrated to monitor the opening and recovery of the blood–brain barrier induced by focused ultrasound using OA/SPECT/CT triple-modality imaging in the mouse model (Zhang et al., 2018a).

### OA/Magnetic Resonance Imaging

**FIGURE 2:**
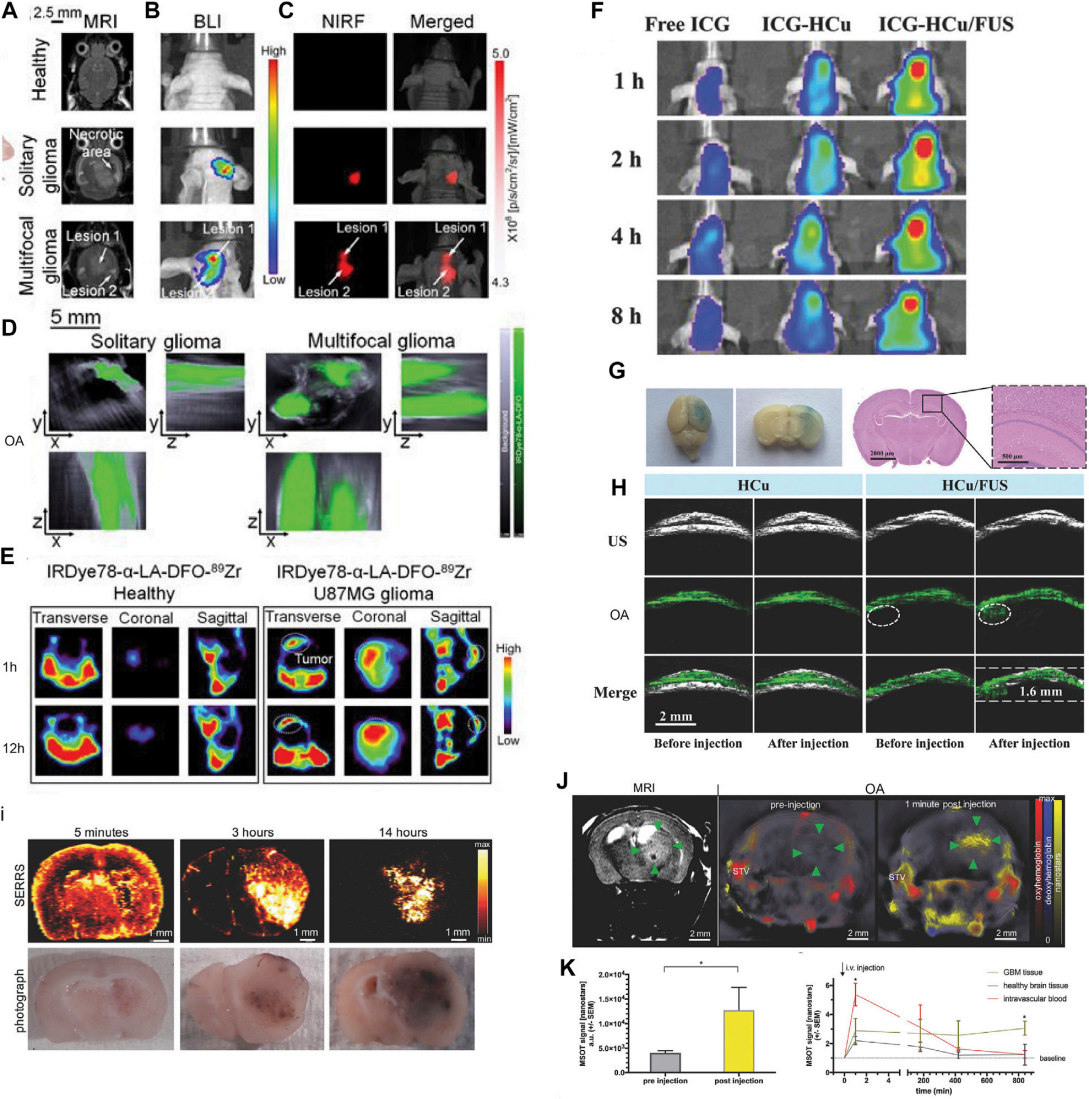
**(A–C)** Imaging of U-87MG orthotopic brain tumors using IRDye78-α-LA-DFO. **(A)** Coronal T_2_-weighted MRI, **(B)** bioluminescence imaging, **(C)** NIR fluorescence imaging, and **(D)** OA imaging of healthy mice and mice with the solitary and bilobed orthotopic gliomas after *i.v.* injection of IRDye78-α-LA-DFO. **(E)** PET imaging of U-87MG glioma after *i.v.* injection of IRDye78-α-LA-DFO[^89^Zr] compared to a healthy control. Reproduced from ref. Yang et al. (2021) with permission from the Ivyspring International Publisher. **(F–H)** Focused ultrasound (FUS)–augmented delivery of biodegradable inorganic hybrid hollow mesoporous organosilica nanoparticles-ss-Cu_2−*x*_Se (HCu) nanosystems for brain tumor detection; **(F)** *In vivo* fluorescence images of U87 glioma‐bearing mice after *i.v*. injection with free indocyanine green (ICG), ICG‐HCu, and ICG‐HCu/FUS; **(G)** Evans Blue and hematoxylin and eosin staining of the mouse brain after FUS‐induced blood–brain barrier opening; **(H)** ultrasound (US), OA, and overlay images of orthotopic brain tumors acquired before and after *i.v.* injection of HCu without or with FUS‐induced blood–brain barrier opening. Reproduced from ref. Wu M. et al. (2018) with permission from the WILEY-VCH Verlag GmbH & Co. KGaA, Weinheim. **(I–L)** Multimodal OA/MRI/Raman imaging using SERRS-MSOT nanostars in glioblastoma-bearing mice. **(I)** The pharmacokinetic profile of the nanostars over the course of 14 h after *i.v.* injection confirmed by *ex vivo* SERRS Raman imaging; **(J,K)** T_2_-weighted MRI and OA imaging showing a significantly increased OA signal unmixed for the nanostars (yellow) in the tumorous area (green arrowheads) and blood circulation (STV, superficial temporal artery and vein, *p < 0.05); **(L)** the peak signal in the blood stream gradually declined over time. No remnant signal deriving from the nanostars was detectable by OA in the blood or the healthy brain tissue area on the contralateral hemisphere. Reproduced from ref. Neuschmelting et al. (2018) with permission from the WILEY-VCH Verlag GmbH & Co. KGaA, Weinheim.

Magnetic resonance imaging (MRI) provides versatile high-resolution structural, functional, and molecular image data with high soft tissue contrast such as T_1_, T_2_ anatomical scans, functional connectivity by using fMRI, white matter integrity assessed by diffusion tensor imaging, blood–brain barrier integrity assessed by dynamic contrast enhanced MRI, cerebral perfusion measured by arterial spin labeling sequence, and molecular imaging using contrast agents (Judenhofer et al., 2008; Ni et al., 2019; Ni et al., 2020c; Massalimova et al., 2021). The structural information derived from MRI helps locate specific molecular information provided by OA tomography after registration. However, the sensitivity of molecular imaging MRI is lower than that of OA imaging and PET. Chen et al. (2015) reported gM-Luc-GRMNBs, multi-theragnostic multi-GNR crystal-seeded magnetic nanoseaurchin, which labeled the injected mesenchymal stem cells in the stroke mouse model for tracking and therapeutic purpose. Many other MR/OA imaging hybrid NPs have been utilized in brain tumor imaging in mouse/rat models, such as Mn^2+^-doped Prussian blue nanocubes, cobalt NPs, PEGylated polypyrrole NPs conjugating gadolinium (Gd) chelates, Gd(III)-phthalocyaninate probes, superparamagnetic iron oxide@Au–labeled stem cells, and copper manganese sulfide nanoplates (Park et al., 2012; Zhang et al., 2012; Gao et al., 2015; Liang et al., 2015; Song et al., 2015; Zhu et al., 2015; Ke et al., 2017; Kim et al., 2017; Qiao et al., 2018; Zhou et al., 2018; Kubelick and Emelianov, 2020; Tomitaka et al., 2020; Yang et al., 2021; Zhang et al., 2021a) (Table 1). Ni et al. (2014) reported ANG/PEG-UCNPs for simultaneous MR/NIR/ upconversion luminescence bimodal imaging target glioblastoma for the efficient tumor surgery. Song et al. (2019) demonstrated using triple-modality MRI/fluorescence/OA imaging probe Fe_3_O_4_@semiconducting polymer NPs for imaging the orthotopic brain U87 tumor mouse model. This NP showed photostability, long-term blood circulation time (t1/2 49 h), and specific tumor uptake (Song et al., 2019). Yang et al. (2021) showed *in vivo* OA/MR/PET/FL imaging using heptamethine sulfoindocyanine IRDye78-α-LA-DFO-[^89^Zr] of glioblastoma in the mouse brain with a low–molecular weight protein alpha-lactalbumin (α-LA) as the carrier to allow efficient hepatic clearance (Figures 2A–E).

### OA/Raman

Few studies have so far utilized hybrid OA/Raman imaging for detection of the molecular and structural changes in the small animal brain. Surface-enhanced resonance Raman (SERS) harbors features such as high sensitivity, brightness, low photobleaching, high resolution, and availability of various tags (Langer et al., 2020). Kircher et al. (2012) demonstrated using an indocyanine green derivative (IRDye-800-c(KRGDf) for triple-modality OA/MRI/Raman imaging of brain tumor detection in the glioblastoma mouse model (Kircher et al., 2012). Neuschmelting et al. (2018) reported OA/SERS dual-modality imaging using SERRS-MSOT-nanostar (absorption peak 770 nm, composed of a gold nanostar core, and encapsulated with IR780-embedded silica layer) for brain tumor delineation in *Nestin-tv-a;Ink4a/Arf* ^*−/−*^ *;Pten* ^*fl/fl*^ glioblastoma mice (Figures 2I–L). Li et al. (2021b) reported ratiometric core-satellite structure AuNNR@MSi-AuNPs for NIR II OA/SERS dual detecting of hydrogen peroxide in inflammation and subcutaneous tumors in the limb of the animal.

### OA/Ultrasound

US imaging is a most frequent combination with OA imaging (Wang and Hu, 2012; Gottschalk et al., 2019; Qian et al., 2019) and provides brain structure information and tumor boundaries, while OA imaging provides molecular or functional readouts (Guo et al., 2017). Encapsulated dye PLGA, methylene blue microbubbles, or nanobubbles have been reported for enhancing the US and OA signals in imaging (Das et al., 2018; Frinking et al., 2020). Santiesteban et al. (2017) showed that copper sulfide perfluorocarbon nanodroplets (CuS–PFCnDs) enhanced contrast in OA/US imaging of the lymph node in mice. Meng et al. (2019) demonstrated US-responsive OA imaging probe Au@lip MBs based on microbubbles (MBs) containing AuNPs for *in vivo* “background-free” OA imaging. In addition, functional US has been developed for imaging microvasculature dynamics at whole brain scale in rodents (Macé et al., 2011; Rabut et al., 2019). Wu M. et al. (2018) reported using focused US-augmented delivery of biodegradable multifunctional inorganic hybrid hollow mesoporous organosilica nanoparticle-ss-Cu_2−*x*_Se (HCu) ICG-HCu for brain glioblastoma imaging and treatment (Figures 2F–H); Park et al. (2021) recently reported using quadruple OA/US/optical coherence/fluorescence fusion imaging with a transparent US transducer for *in vivo* monitoring of rat eyes after injuries.

### OA/Computed Tomography

CT is widely used for providing structural information in combination with PET, SPECT, or fluorescence imaging studies in small animals (Polatoglu et al., 2019; Herfert et al., 2020). A few studies have been reported using NPs such as porous MnO@Au nanocomposites and Pdots@hydrogel nanoplatform for MR/OA/CT tumor imaging in the peripheral (Liu et al., 2018; Men et al., 2020). A recent study by Shang et al. (2017) demonstrated an OA/CT/MRI triple-modality core-shell Au nanorod@metal–organic NP for imaging U87MG gliomas in mice with low toxicity, strong X-ray attenuation, and high contrast and penetration depth. Lv et al. (2019) showed OA/CT/upconversion luminescence/short-wavelength infrared luminescence imaging using a UCNP@mSiO_2_-ZnPc NP (using NaErF_4_ as host) for brain glioblastoma imaging. Biocompatible conjugated polymer nanoparticles for highly efficient photoacoustic imaging of orthotopic brain tumors in the second near-infrared window. The CT intensity and the OA signal intensities correlated with different concentrations of this NP with a high signal-to-noise ratio.

## Discussion

The multiplex molecular, structural, and functional imaging readouts using OA imaging provide important etiological insights into brain function and disease pathophysiology in small animal models. There is a rapid development in molecular imaging contrast agents employing a multimodal imaging strategy for pathological targets involved in brain diseases. Hybrid imaging systems such as SPECT/PET/CT and PET/MRI have greatly improved the workflow and data analysis (Lancelot and Zimmer, 2010; Rodriguez-Vieitez et al., 2015). Fluorescence imaging/MRI hybrid imaging enables to answer the longstanding research questions such as the link between MRI blood-oxygen-level-dependent readout and calcium recording (Schulz et al., 2012; Schlegel et al., 2018; Lake et al., 2020). We propose the following aspects of particular interest for the development in small-animal OA hybrid brain imaging.

1) Registration and analysis: In most small-animal OA imaging studies, the data from different imaging modalities were acquired sequentially (Ni et al., 2018c). Co-registration and post-processing of small-animal neuroimage datasets acquired sequentially using OA imaging and other modalities have been performed for the region/volume of interest analysis (Attia et al., 2016; Ren et al., 2019). For this, manual/semi-automatic atlas–based analysis and algorithms have been developed (Ren et al., 2019; Ren et al., 2021; Zhang et al., 2021b). Further studies to develop a deep learning–based method for fully automatic segmentation and registration are needed, for example, between OA/MRI or OA/CT brain imaging data and for position-dependent light fluence correction hold great promise (Sarah et al., 2019; Waterhouse et al., 2019; Ni et al., 2020a; Dean-Ben et al., 2020; Hu et al., 2021). Additionally, bimodal animal holder (Gehrung et al., 2020; Zhang et al., 2021b) or concurrent imaging acquisition OA tomography–MRI, OA–fluorescence confocal microscopy, and OA tomography–fluorescence imaging have already been developed (Chen et al., 2017; Zhang et al., 2018b; Liu et al., 2019b; Ren et al., 2021; Zhang et al., 2021c; Dadkhah and Jiao, 2021; Deán-Ben et al., 2021). Further development in synchronized OA-MR platforms for small-animal brain imaging for simultaneous detection will further improve the workflow (Ren et al., 2021).
2) Modeling of pharmacokinetics: One compartment fluence independent model has been reported for OA imaging in the tumor tissue of the animal model (Hupple et al., 2018). So far, no kinetic modeling has been developed and validated for OA brain imaging. For the OA tomographic imaging data, pharmacokinetic modeling will facilitate the interpretation of results more and improve accuracy and the further development of imaging probes.
3) Standardization: Various aspects can impact on *in vivo* OA imaging data quality in small animals, such as an imaging protocol, anesthesia and animal handling, OA signal calibration, an image analysis method, and data processing and sharing tools. Standardization on the phantom OA imaging data has been initiated (Bohndiek et al., 2019), and further image acquisition and post-processing regarding small animal brain imaging data are essential.
4) New multimodal NIR II probes: There is a rapid development in hybrid OA imaging probes, especially NIR II probes for brain imaging. NIR II probes allow for deeper penetration, improved signal/noise ratio, and more reliable unmixing from strong endogenous hemoglobin background. Many NIR II OA/fluorescence probes, such as novel NIR II OA probes with DNA-based nanocarriers, PEGylated Au nanoparticles, and SPNs, that are of high chemical stability, low toxicity, and a high signal-to-noise ratio showed great promise for multimodal imaging and photothermal therapy (Jin et al., 2010; Ding et al., 2019; Meng et al., 2019; Sun et al., 2019; Zhang et al., 2019c; Feng et al., 2020; Xu et al., 2020; Joseph et al., 2021; Miyasato et al., 2021).
5) Toward clinical translation: For fluorescence imaging, the U.S. Food and Drug Administration (FDA) approved several probes such as ICG (Mokrousov et al., 2021), methylene blue, fluorescein, Prussian blue, 5-aminolevulinic acid (Stummer et al., 2006), and Evans blue. A few fluorescence imaging contrast agents are in clinical trials at different 0stages including ONM-100 (pH-activable NP), second window ICG or SWIG, BLZ-100, Tumor Paint™, TumorGlow™, ABY-029, LUM015, SMG-101, OTL38, and Cornell dots (Phillips et al., 2014; Whitley et al., 2016; Gutowski et al., 2017; Randall et al., 2019; Samkoe et al., 2019; Wahsner et al., 2019; Voskuil et al., 2020; Teng et al., 2021). For MRI, eight Gd-based probes such as Gd-DOTA and superparamagnetic iron oxide agents, such as ferumoxytol, ferucarbotran, and ferumoxtran‐10 (Combidex/Sinerem) have been approved by the FDA. For US imaging, Definity (perflutren lipid microspheres), Optison (human serum albumin stabilized perflutren microspheres), SonoVue (phospholipid-stabilized microbubble), and Sonazoid (F‐butane encapsulated in a lipid shell) have been approved by the FDA for clinical usage; further clinical studies with OA imaging contrast agents are needed. Applications of OA imaging in the clinical research have shown promising results mainly in the peripheral with endogenous contrast (melanin, Hb, and HbO) such as in inflammatory bowl, dermatology, and breast cancer (Garcia-Uribe et al., 2015; Knieling et al., 2017; Masthoff et al., 2018; Nyayapathi et al., 2021). Na et al. (2021); Na and Wang (2021) recently demonstrated the first OA imaging in a living human brain. Significant challenges need to be overcome for OA human brain imaging due to the thickness of the human skull, the acoustic distortions, and penetration depth.

To conclude, multimodal OA brain imaging assisted with contrast agents in small animals has facilitated the understanding of brain physiology and disease-related mechanisms. As OA imaging is a rapidly evolving technique, many outstanding challenges need to be tackled to further improve the quantitativeness and achieve even wider applications.
